# Evidence to Action: Translating Innovations in Management of Child and Adolescent TB into Routine Practice in High-Burden Countries

**DOI:** 10.3390/pathogens11040383

**Published:** 2022-03-23

**Authors:** Brittany K. Moore, Riitta A. Dlodlo, John Paul Dongo, Sabine Verkuijl, Moorine P. Sekadde, Charles Sandy, Susan A. Maloney

**Affiliations:** 1 Division of Global HIV and TB, U.S. Centers for Disease Control and Prevention, Atlanta, GA 30329, USA; szm7@cdc.gov; 2 Department of Tuberculosis, The International Union Against TB and Lung Disease, Zimbabwe Office, Bulawayo 029, Zimbabwe; rdlodlo@theunion.org; 3 Department of Tuberculosis, The International Union Against TB and Lung Disease, Uganda Office, Kampala P.O. Box 16094, Uganda; jpdongo@theunion.org; 4 Global Tuberculosis Programme, World Health Organization, 1202 Geneva, Switzerland; verkuijls@who.int; 5 National TB and Leprosy Programme, Kampala P.O. Box 16069, Uganda; moorine.sekadde@gmail.com; 6 National TB Control Programme, Harare 242, Zimbabwe; dr.c.sandy@gmail.com

**Keywords:** tuberculosis, pediatric, adolescent, partnership, decentralization, advocacy

## Abstract

Child and adolescent tuberculosis (TB) has been long neglected by TB programs but there have been substantive strides in prioritizing TB among these populations in the past two decades. Yet, gaps remain in translating evidence and policy to action at the primary care level, ensuring access to novel tools and approaches to diagnosis, treatment, and prevention for children and adolescents at risk of TB disease. This article describes the progress that has been made and the gaps that remain in addressing TB among children and adolescents while also highlighting pragmatic approaches and the role of multisectoral partnerships in facilitating integration of innovations into routine program practice.

## 1. Introduction

**Figure 1:**
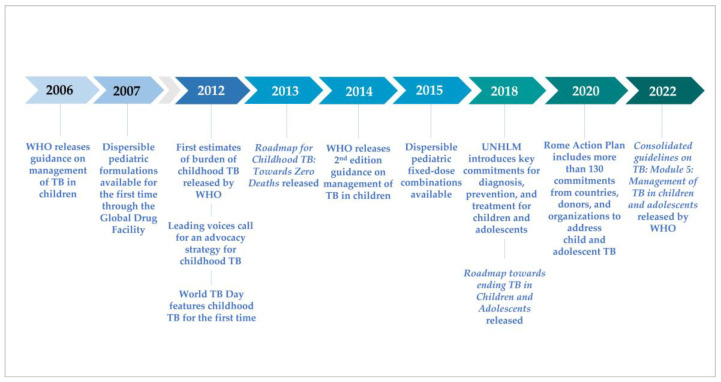
Timeline of key events in advocacy and multilateral action for child and adolescent TB.

After nearly 40 years with few novel diagnostic tools or anti-tuberculosis (TB) drugs, the past decade has been one of accelerated innovation on all fronts for TB. During this time, the global TB community finally recognized and began to address the unique needs of children with TB (Figure 1). The first comprehensive global guidance on the management of TB in children was released by WHO in 2006 [1], followed in 2007 by availability through the Global Drug Facility (GDF) of (limited) dispersible pediatric formulations for high-burden countries. The childhood TB subgroup was established in 2003 under the DOTS expansion working group of the Stop TB Partnership, with the secretariat hosted by the World Health Organization’s (WHO) Global Tuberculosis Programme, comprised of clinicians, researchers, academics, experts, and donors who developed a comprehensive advocacy strategy to address TB among children [2]. In 2012, the first estimates of the burden of childhood TB published by WHO revealed the burden to be much greater than anticipated [3,4], World TB Day focused on children for the first time, and the Stop TB Partnership launched their advocacy effort, “No more crying, no more dying.” The childhood TB subgroup coalesced around key priorities for research, development, policy, and programming in 2013 with the release of the Roadmap for Childhood TB: Toward Zero Deaths [5]; the Guidance for national tuberculosis programmes on the management of tuberculosis in children was published in 2014; and partnerships and communities of practice were established across disciplines and health programs to support implementation of important advances and innovations.

Joint priorities, commitments, and targets have been established in the years since by the WHO, national TB programs, and donor institutions. The childhood TB subgroup gained full implementation working group status in 2017 and expanded to include a focus on adolescents, becoming the Child and Adolescent TB Working Group in 2017. In 2018, the United Nations (UN) held its first High-Level Meeting on the Fight Against Tuberculosis (UNHLM), announcing a political declaration including targets for TB diagnosis, treatment, and prevention in children [6]. The second edition of the Roadmap (Roadmap towards ending TB in children and adolescents) [7] was released alongside the UNHLM, indicating key actions necessary to reach the Declaration’s ambitious goals. In 2020, the Vatican added pediatric TB to the high-level dialogues for HIV resulting in the development of the Rome Action Plan, which includes a large number of commitments to address pediatric TB [8]. Additionally, in 2022, the WHO will release updated guidelines with an operational handbook on the management of TB in children and adolescents, providing critical updates on novel diagnostic approaches, drug regimens, and models of TB care that the global health community can translate into routine program practice [9].

While progress towards these goals has been significant in the last decade, recent gains have been undermined by the COVID-19 pandemic. Between 2019 and 2020, there was an 18% and 24% reduction in TB case notifications overall and among children, respectively, and an overall increase in TB-related mortality for the first time in a decade [10]. Major and disproportionate gaps remain in addressing TB among children and adolescents and in translating available tools and approaches into nation-wide services [7]. Later-stage translation of evidence to action often lags for children and adolescents, with adaptations or extrapolations following only after advances are available for adults. The global TB community is positioned to integrate and scale up these innovations, building on a growing multisectoral movement comprised of national programs, multisectoral institutions, parliamentarians, as well as private, non-profit, and academic partners. This paper will outline the key remaining gaps in the TB clinical cascade for children and adolescents, the indispensable multisectoral partnerships needed for progress, and examples of translation of evidence to action to the most important juncture: where the child meets the health care worker.

## 2. Gaps in the TB Care Cascade for Children and Adolescents

**Figure 2:**
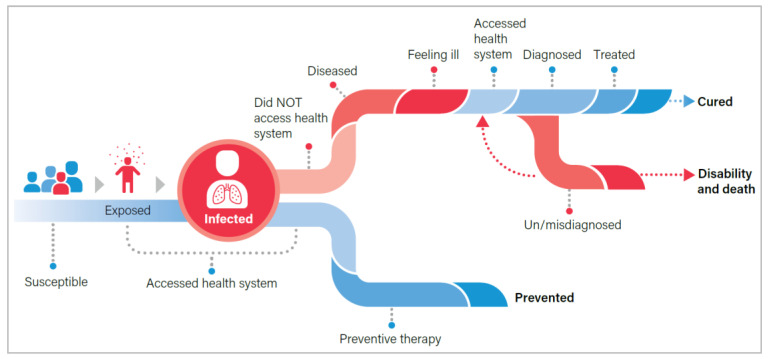
Pathway through TB exposure, infection, and disease from the Roadmap towards ending TB in children and adolescents [7].

In 2020, an estimated 1.1 million children (aged <15 years) developed TB (11% of the burden), while nearly 226,000 died from this preventable, curable disease (16% of all deaths) [10]. It is estimated that 80% of child deaths from TB are among children under five, while 95% are among those who never received treatment [11]. This disproportionate burden underscores the unique vulnerability of young children, particularly a higher likelihood of rapid progression from infection to severe manifestations of TB disease [12,13,14,15,16] and highlights the challenges in providing health services to this vulnerable group [17,18,19]. Adolescents face other unique challenges when accessing TB services and may require greater psychosocial and peer support as well as alternative models of care to accommodate school or other obligations. The WHO Roadmap towards ending TB in children and adolescents describes the prevention, detection, and treatment gaps (Figure 2), where children and adolescents do not receive the services necessary to prevent or cure TB [7].

### 2.1. Prevent

Given the challenges and gaps in case detection, preventing TB disease among at-risk children and adolescents before they become ill is a priority intervention [20,21,22]. When a person is exposed to TB, bacteria can enter the body. If the immune system can contain the bacteria, that person may not develop active TB disease but will remain in an infected state, a condition known as TB infection. Most people who become infected will never develop disease, but if the person has an immature or compromised immune system—as in the case of young children or people living with HIV (PLHIV)—they can progress from infection to active disease quickly. TB preventive treatment (TPT) treats the infection and prevents progression to active disease. Since commitments to scale up TPT were made at the UNHLM in 2018 [6], the number of eligible people initiating TPT across all categories has doubled year on year [19]. However, after three years, only 29% of the UNHLM target to provide TPT to 4 million child household contacts aged under 5 years and less than 2% of the target to provide TPT to 20 million contacts 5 years and over by 2022 has been reached [10]. The target to provide TPT to six million PLHIV is the only one that has been reached: 7.2 million PLHIV had received TPT between 2018 to 2020 [10]. The U.S. President’s Emergency Plan for AIDS Relief (PEFPAR) committed to provide TPT to all PLHIV in PEPFAR-supported programs, and from 2018 to 2021 more than 10 million PLHIV have initiated TPT [6,23]. During this time, nearly 75% of the 650,000 children living with HIV (CLHIV) receiving care through PEFPAR-supported programs have initiated TPT with an average 84% completion rate [23]. Contact investigation is a powerful strategy to identify children with TB as well as others who will benefit from TPT [24]. After several years of improving implementation of contact investigations, both contact investigation and initiation of TPT declined in 2020 due to the COVID-19 pandemic, undermining gains in prevention [10].

There are additional gaps in safety and efficacy data for use of new, shorter TPT regimens among children under 2 years and CLHIV, such as 12 weekly doses of isoniazid and rifapentine (3HP), delaying access to preferred, more effective regimens [25,26]. Data on efficacy, safety and pharmacokinetics for this regimen are expected from an ongoing randomized clinical trial in 2022 [27]. The shorter-course 3-month daily isoniazid and rifampicin (3HR) regimen, using existing fixed-dose pediatric formulations of HR, can be implemented for child contacts and some CLHIV (e.g., on efavirenz (EFV)-based ART) now while awaitingtrial results on the use of 3HP and 1HP in children, though most CLHIV have been transitioned off EFV-based regimens. Despite these limitations, the primary barriers to scaling up TPT among children and adolescents remains a lack of political will and human resources for health (HRH) [20].

### 2.2. Find

While case notifications of TB among children have nearly quadrupled in the past decade, only 45% of all children (<15 years) and 28% of young children (<5 years) with TB were diagnosed in 2020 compared with 62% of adults [10]. Among the estimated 25,000–32,000 children who develop drug-resistant TB every year [28], only 10% were diagnosed [10]. Unfortunately, notifications and treatment initiations have declined since 2019 for all ages due to service disruptions caused by the COVID-19 pandemic [10]. There are unique differences in the clinical presentation of TB in children (e.g., higher likelihood of paucibacillary or extrapulmonary disease, non-specific symptoms, unique features on chest radiograph, and difficulty producing sputum) that impede progress in detection and bacteriological confirmation of childhood TB [9,13,14,29,30]. Diagnostic tests, including molecular rapid diagnostics, smear microscopy and culture, have limited sensitivity among children because of these unique clinical features. There are also differences in the ways children enter the health system, often through well-child, immunization, or nutrition clinics, that may delay evaluation for TB. A series of innovations in screening [31], diagnostic tests [32], and use of child-friendly specimens on existing platforms [9] may address some of these issues. However, validation of novel diagnostic tests among children has been slow, following only after approval and use among adults, and dedicated training and mentorship on clinical diagnosis and management of TB in children is often overlooked. Considering the underlying challenges of microbiological confirmation of TB in children, health care worker experience and confidence in making a clinical diagnosis remain critical and must be strengthened alongside implementation of new technologies. These unique elements require adaptation of diagnostic methods and networks, introduction of techniques specifically developed for children, community awareness and education on child TB for caregivers, decentralization of screening beyond conventional adult services, and training for clinicians, community health workers, and volunteers. 

### 2.3. Cure

Most children respond well to preventive and curative treatment regimens for drug-susceptible and drug-resistant TB and experience fewer and less severe side effects than adults [15,16,17,20,30]. Historically, pediatric TB regimens have been based on extrapolations from adult regimens, often requiring crushing tablets and estimating dosing for children [33,34]. However, currently, paediatric trials on TB regimens are being conducted, and the recently completed SHINE trial on a shorter regimen to treat drug susceptible non-severe TB has informed updated WHO guidelines. There are differences in bacillary burden, type and site of disease, and metabolism that affect bioavailability and distribution of anti-TB drugs in children, but there still have been relatively few pre-clinical models and clinical trials to identify optimal dosing, regimen composition, and formulations for children [34,35,36,37]. Substantial lags in the development of taste-masking, dispersible formulations in child-appropriate dosages remain—lags that range from six to 13 years following availability of adult formulations [17,35,38,39,40]. Still, advances in the development of novel preventive treatment regimens and drugs for treatment of drug-resistant TB have been made in the last decade, with growing coalitions developing pre-clinical models for designing pediatric regimens and bringing pediatric formulations of novel drugs to market [35,36,41]. Efforts to rollout these new formulations should focus in the near-term on providing access to Ministries of Health through negotiated, consolidated procurement, training health care workers to implement, and incorporating regimens into routine practice. Beyond implementation of novel formulations and regimens, innovations in service delivery and decentralized models of care should also be a priority, providing family-centered, flexible options for children and their families.

### 2.4. Sustain

Existing gaps in the care cascade underscore how hard-won innovations in management of child and adolescent TB encounter barriers at the implementation level. While historical neglect of childhood TB has been well-documented [7,12,13,15,38], we have entered a period with new tools and approaches amid a growing consensus that TB among children and adolescents should be prioritized [6,9,42]. Introduction and implementation of any innovation into routine service requires political will, funding, adaptation to local context, training, and careful monitoring and mentorship. The neglect of child and adolescent TB is still evident in their lack of inclusion in national strategic plans, guidelines, costed annual TB program budgets, and funding proposals to donors. This leads to chronic under-funding of programs providing services to these populations, including governance mechanisms (e.g., child and adolescent TB technical working groups), commodities, training, and HRH—all critical elements to ensure sustainable translation of innovation into routine practice. Communities of Practice and other platforms for advocacy and information sharing can raise awareness of these existing gaps and press for prioritization of child and adolescent TB. The authors are part of such platforms, the Child and Adolescent TB Working Group, and the Union/CDC Sub-Saharan Africa Regional Child and Adolescent TB Centre of Excellence (COE) which aims to raise awareness, strengthen governance, build capacity, and enhance adaptation and local action for child and adolescent TB with partner national TB programs (NTP). Recent innovations can be implemented at scale to begin closing gaps in diagnosis, treatment, and prevention but it will require enhanced, intentional efforts to translate evidence to action at all levels.

## 3. Evidence to Action through Multisectoral Partnerships

The new WHO consolidated guidelines [9] and operational handbook [43] on the management of tuberculosis in children and adolescents represent the next step in implementing nearly a decade of work to develop novel diagnostic methods, treatment regimens, and service delivery approaches for children and adolescents. The guidelines and handbook reflect the latest evidence and implementation experiences and were informed by engagement with a multidisciplinary team of experts (including civil society)—critical components in a dynamic continuum to integrate innovation into routine practice [44]. There are many ways to frame this continuum: we provide one such conceptualization (Table 1) to describe the many components required to translate evidence to action in broad categories, including agenda setting, innovation inception and delivery, program implementation and continuous quality improvement, and building political will. This table also demonstrates the iterative nature of development and adaptation of innovations, which involves stakeholders at the multilateral, national, and community level working together to ensure new evidence can be implemented routinely at scale and is centered around patients’ needs. We will explore two examples in more detail to illustrate the engagement required at all levels from global to local: (a) development of child-friendly formulations and (b) decentralizing care through adaptations in service delivery.

### 3.1. Bench to Bedside: Development and Uptake of Child-Friendly First-Line Drug Formulations

In 1994, the WHO recommended standardized short-course multidrug regimens for treatment of drug-susceptible TB in adults, including six to eight months of a combination of rifampicin (R), isoniazid (H), and pyrazinamide (Z) [45]. This was followed in 2003 by the recommendation of the now-standard regimen including two months of RHZ plus ethambutol (E) followed by four months of RH [46]. It was not until 2010 that the WHO released rapid guidance on TB treatment in children following clinical trials to establish appropriate dosing strategies in children [47]. However, the fixed-dose combination (FDC) formulations available were not palatable or compatible with new dosing guidelines. For more than 20 years, dosing for children required extrapolations, estimations, and cutting and crushing tablets formulated for treating adults, complicating efforts to ensure children were receiving therapeutic doses of anti-TB drugs and burdening health care providers and caregivers. New dosing strategies paired with global burden estimates paved the way for research and development into child-friendly FDCs for treatment of drug-susceptible TB. In 2012, Unitaid provided an initial grant—alongside support from aid agencies in the United States, United Kingdom, Australia, and the Netherlands—to The Global Alliance for TB Drug Development (TB Alliance) to establish the Speeding Treatments to End Pediatric TB (STEP-TB) project to catalyze development and uptake of pediatric TB formulations [33,48].

This ambitious project sought to develop a child-friendly, dispersible formulation while also ensuring rapid uptake and adoption along with a sustainable, stable marketplace. Early partnerships were established among academics, governments, non-government organizations, and the pharmaceutical sector to ensure support for the regulatory approval, procurement, and uptake of these formulations [33,48,49]. Partnering with Macleods and academic and technical institutions, STEP-TB developed a water-soluble, palatable FDC based on the dosing strategies recommended by the WHO and released in their 2014 updated guidelines [29]. In October 2016, this formulation was introduced in Kenya alongside the “Louder than TB” campaign, a local movement to address TB among children [50]. By March 2017, the WHO had pre-qualified the formulation and included it in the Essential Medicines List for children (EMLc) enabling the Stop TB Partnership GDF to make these formulations available for procurement [33]. TB Alliance, the WHO, and other partners offered technical support for regulatory approval and strategies for uptake [49] and the Global Fund to Fight AIDS, TB, Malaria (GFATM) encouraged inclusion in funding proposals. By May 2017, 20 high-burden countries had strategies in place for introducing formulations, including engaging communities and caregivers and updating guidance and supply chain mechanisms. Since 2017, 116 countries have procured more than one million courses of this FDC formulation [51].

This success demonstrates the importance of multisector engagement, buy-in, and joint prioritization and planning from the earliest stages through scale up. More recently, many of these same partners have used similar approaches to catalyze development of pediatric formulations of bedaquiline and delamanid [52], including listing them in the 8th WHO Essential Medicine List for Children [53] and including interim dosing strategies in the new operational handbook. Unfortunately, safety, efficacy, and dosing studies for children are still not conducted in parallel with adult studies for new drugs, and some existing regimens still have no appropriate dosing strategy for children. One proposed approach highlighted at the 52nd Union World Conference on Lung Health to shorten the time to market for appropriately dosed child-friendly formulations is called Chasing Expedited and Equitable Treatment Access for Children (CHEETA). CHEETA hopes to establish an umbrella platform and protocol focused on expeditious investigation of new compounds and dose-optimization for existing drugs for children [40]. Such bold coalitions can potentially overcome the delays in this sector.

### 3.2. Decentralizing TB Services for Children and Adolescents: Operational Research and Program Adaptation

The most important player in bringing an innovation to scale is the NTP: establishing priorities and funding obligations, developing and maintaining the systems to deliver and monitor critical health services, always with an eye on integrating innovations and best practice. NTPs have been rapidly adapting to COVID-19 by scaling-up decentralized models of care, but interest in diversifying service delivery options had been growing for years. Person-centered care is being prioritized in TB [54], HIV [55], and other health programs worldwide. Decentralizing diagnosis and treatment services are cornerstones to this new paradigm, but operationalizing this approach is highly context-dependent and requires adaptation through operational research.

A systematic review presented at the 52nd Union Conference explored more than 25 studies across Asia, Africa, and South America assessing decentralized diagnosis, treatment, and/or prevention services for children and adolescents [56] demonstrating both the interest in developing scalable options and the heterogeneity of the approaches. The Elizabeth Glazer Pediatric AIDS Foundation’s (EGPAF) Catalyzing Pediatric TB Innovations (CaP-TB) project—which was part of this systematic review—has been implementing and evaluating decentralized approaches for both case detection [57] and TPT [58] across multiple Sub-Saharan African countries and India, demonstrating increased case detection as well as TPT initiation and completion with variations by country. PEPFAR, which has been scaling-up differentiated service delivery approaches for HIV care and treatment, recently released considerations for incorporating TPT into these services for children and adolescents [59].

Another project included in the systematic review was the Decentralize TB services and Engage Communities to Transform lives of Children with TB (DETECT TB) project which was implemented in Uganda from 2015 to 2016. The National TB and Leprosy Program (Uganda NTLP) worked with the International Union Against Tuberculosis and Lung Disease (The Union) to test a sustainable, decentralized health systems delivery model for child and adolescent TB. Initially piloted in only two districts, the project aimed to assess the feasibility of decentralizing childhood TB case detection, diagnosis, treatment and prevention from tertiary to primary health care facilities [60,61]. Health care workers at the primary care level were trained on management of TB among children (<15) with an emphasis on clinical diagnosis, provided a performance-based stipend to conduct household TB contact screening, referral, and treatment follow-up, and received on-site mentorship and supportive supervision [62]. Overall, case detection nearly doubled, TB treatment completion increased 17%, and TPT initiation among eligible children skyrocketed from 5% to 77% [61].

Following this successful pilot, the NTLP in collaboration with partners, refined its approach to child TB activities in its 2015–2020 and 2020–2025 National Strategic Plans, including national household contact investigation activities and screening tools. In 2018, the NTLP secured funds through the Global Fund to scale up this model to 10 poorly performing districts, ultimately increasing child TB case notification by 27% [61]. The Global Fund has awarded additional funding under the new funding model to sustain implementation in initial scale-up districts and expand to 40 new districts, resulting in 50 (37%) districts in Uganda implementing the approach [63]. This iterative and stepwise scale up also illustrates the elements needed for sustainability: refinement of a proven approach that can be integrated into national policy, incorporating the activities into priorities, budgets, and funding proposals, and providing the human and financial resources necessary for expansion.

Across these examples, successful approaches often incorporated interventions at multiple levels in the community and health system, included initial and follow-on training, and emphasized supportive supervision and mentorship [56,57,58,61,63]. There are several excellent examples that illustrate more directly other elements in the continuum described in Table 1, especially those that focus on use of routine data for decision making and supportive supervision and mentorship, which go hand-in-hand for continuous quality improvement. The work in Zimbabwe [64], Zambia [65], and Benin, Burkina Faso, Cameroon and Central African Republic [66] to use data in real time to address TB program gaps and performance issues could be applied to close gaps in care for child and adolescent TB.

## 4. Evidence to Action: A Way Forward

Bringing innovations to scale requires establishing common goals and priorities among key stakeholders while also coordinating implementation and scale up at all levels of the health system. The UNHLM Declaration has established common goals and commitments, emphasized in the Roadmap and the Rome Action Plan, and there is growing interest in greater coordination across stakeholders. However, gaps remain, and more must be done at all levels to ensure that we do not fail in bringing the latest innovations to the people who need them most.

Several actions could be prioritized in agenda setting, innovation inception and delivery, continuous quality improvement, and building political will to accelerate delivery of innovations to the primary care level (Table 1). Ministries of Health and NTPs can explicitly incorporate child and adolescent TB prevention, screening, diagnosis, and treatment into national strategic plans, guidelines, budgets, and funding proposals while also establishing and empowering child and adolescent TB technical working groups to develop strategies and coalitions for implementation. Similarly, multilateral and donor institutions could prioritize clear budgeting for child and adolescent TB activities which would ensure appropriate funding for priority actions. Further, in developing strategies for child and adolescent TB, all stakeholders could move toward differentiated care approaches with tailored services for different age groups to ensure age-specific challenges are being addressed. These institutions and governments should continue to advocate for and coordinate to ensure equitable access to novel diagnostics and treatment options through platforms such as the Global Drug Facility.

Academic institutions and clinical trials consortia can support parallel enrollment of children and adolescents in clinical trials and validation studies of novel diagnostics and treatments with standardized age categories to reduce time to implementation. Similarly, academic, public-, and private-sector partners can deepen investment in research and development for novel vaccines and tests of TB infection to accelerate innovation.

Given the importance of developing clinical skills and confidence in diagnosis and treatment of child and adolescent TB, capacity building strategies that emphasize skill transfer and development at peripheral levels of the health system are critical to ensuring integration into routine practice. At sub-national and national levels, data-driven mentorship aimed at quality improvement can enhance skill transfer while establishing a mechanism for shared problem-solving. Communities of Practice play an important role at all levels, providing platforms for testing new approaches and sharing information and best practices. Involving community and civil society organizations in oversight and service delivery design at all levels can also ensure care is responsive to individual and community needs while encouraging health-seeking behavior. Organizations can mobilize resources for this work through national and donor organizations, such as the Stop TB Partnership’s Challenge Facility for Civil Society.

The COVID-19 pandemic has also demonstrated that health systems can quickly overcome monumental challenges if there is a will to do so. The mechanisms and platforms established to address the pandemic can be used to close remaining gaps, capitalize on established multisectoral partnerships, and expedite the translation of evidence to action. Coordination across national programs, including TB, HIV, and maternal child health services would help ensure consistency of training and integration of TB into service delivery platforms accessed by children and their families. All partners working together would help ensure wide-scale implementation of the new evidence-based approaches from the child and adolescent TB guidelines.

The global TB community knows what success looks like. Now comes the hard work of coordinating action to ensure the latest innovations and evidence-based practice are accessible to the children and adolescents who need them most.

## Figures and Tables

**Table 1:** Core Elements in the Translation of Evidence to Action.

Core Elements	Selected Examples of Recent Progress	Way Forward
**Agenda Setting and Normative Guidance**		
**Establishing Research and Development Priorities:** Identifying the greatest need for novel approaches, tools, and regimens to improve care	WHO Roadmap towards ending TB in children and adolescents WHO Pediatric Anti-TB Drug Optimization (PADO-TB) ➢ Stop TB Partnership Child and Adolescent TB Working Group establish pediatric formulations as a priority	Establish more public–private partnerships to accelerate action on identified priorities
**Policy and Guideline Development:** Synthesizing latest evidence, identifying priority interventions, establishing reference standards and benchmarks	WHO Consolidated guidelines on TB. Module 5 Management of TB in children and adolescents National TB Guidelines ➢ 2010 WHO Rapid Advice: Treatment of TB in Children; 2014 Guidance for national TB programs on the management of TB in children ✓ 2017 WHO Guidelines for the treatment of drug-susceptible TB and patient care establishes person-centered care as a priority for NTPs ✓ CaP-TB and DETECT projects inform 2022 WHO child and adolescent TB guidelines	Adapt global guidance to national context through coordination with MOH, technical, and funding partners
**Establishing Implementation Priorities:** Establishing program priorities for implementation	National TB Strategic Plans and budgets Global Fund to Fight AIDS, TB and Malaria (GFATM) prioritizes children under 5 for TB funding proposals (2020) ➢ NTPs work with the WHO, STP Global Drug Facility (GDF), TB Alliance to prioritize child-friendly FDCs ➢ Unitaid and donor organizations from several nations catalyze creation of STEP-TB ✓ Uganda NTLP incorporated decentralized care into successive National Strategic Plans (2015–2025) and funding proposals to GFATM	Include child and adolescent TB interventions in NSP, costed budgets, and funding proposals Donors prioritize child and adolescent TB in requests for proposals
**Operational and Implementation Guidance:** Developing tools, algorithms, job aids, training materials, and other practical guidance required for implementation	The WHO operational handbook accompanying guidelines STP Child and Adolescent TB Working Group (CAWG) develop budgeting tools to improve costing of child and adolescent TB interventions WHO releases Best practices in child and adolescent tuberculosis care (2018) Implementation guidance for use of non-sputum specimens for diagnosis of TB among children ➢ The WHO releases Technical Briefing Note: Technical step process to switch to new pediatric TB formulations; TB Alliance and others offer technical support for regulatory and training needs ✓ Uganda NTLP adapts approach, training, and tools for expanded scale up beyond pilot districts	Build upon the WHO Operational Handbook implementation guidance to offer context-specific tools and adaptations
**Innovation Inception and Delivery**		
**Pre-Clinical Development and Clinical Trials:** Trials establishing safety, efficacy, non-inferiority of novel treatment regimens or diagnostic techniques	TAG Pipeline Report TB Alliance: New Pathways for Childhood TB Treatment Clinical trial networks (e.g., IMPAACT4TB, TBTC, DOLPHIN KIDS) ➢ Macleods develops water-soluble FDC of RHZ and RH ➢ Macleods with Baylor and Stellenbosch Universities and TB Alliance conduct trials of the new FDC	Increase investment for TB R&D in accordance with UNHLM targets Develop pre-clinical dosing models in children and include children in parallel with adults to speed access to new tools
**Regulatory Approval:** International and national	➢ TB Alliance and partners work with the WHO to achieve pre-qualification and listing on the EMLc and provide technical support to NTPs for regulatory approval	Prioritize review for pre-qualification of pediatric formulations
**Market Shaping and Consolidated Procurement:** Quantifying market for therapeutics, diagnostics, commodities; consolidated procurement with negotiated prices	STP/Global Drug Facility WHO PADO ➢ STP/GDF work with TB Alliance and MacLeods to bulk procure product upon agreement with NTPs for forecasted needs; WHO Technical Brief provides forecasting advice	Expand pooled procurement Incentivize manufacturers of pediatric TB medications through improved stakeholder coordination Streamline regulatory approvals Increase consumer education on guidelines
**Program Implementation and Continuous Quality Improvement**		
**Surveillance and Data for Decision Making:** Routine recording and reporting of indicators paired with routine use of data and experience to improve performance by targeting gaps and developing solutions	Making sense of TB data (Zimbabwe) TB Situation Room (Zambia) Clinic-Lab Interface for Continuous Quality Improvement (CDC CLICQ!) ➢ NTP and TB Alliance monitor proportion of patients receiving new FDC in early rollout; adverse event and discontinuation monitored over time ✓ Uganda NTLP monitored expansion of decentralized care approach through routine surveillance in 10 expansion districts	Ensure appropriate fine age disaggregation at all levels to assess performance across age groups
**Training TB Program Staff and Health Care Workers:** Providing routine training to all health cadres and volunteers involved in delivery of health services	The WHO TB knowledge-sharing platform TB-Speed Project Extension for Community Healthcare Outcomes (ECHO) ➢ Health care workers and caregivers trained on administration of new FDC ✓ Catalyzing Innovations for Pediatric TB (CaP-TB) and Decentralize TB services and Engage Communities to Transform lives of Children with TB (DETECT TB) projects emphasize training at the primary care level	Develop capacity building strategies that emphasizes skill transfer at the lowest levels of health system
**Supportive Supervision and Mentorship:** Providing oversight against performance targets and mentorship for skill development	TB Situation Room (Zambia) ✓ CaP-TB and DETECT TB provided on-site supervision and mentorship to sustain skill development	Integrate routine mentorship to improve performance at lowest levels of health system
**Operational Research and Program Adaptation:** Adapting evidence-based innovations to a specific context through implementation and evaluation	Clinical trial and operational research consortia (IMPAACT4TB, CaP-TB, TB-Speed) STP TB REACH ➢ Louder than TB Campaign in Kenya adapts the use of formulation to Kenyan context; IEC materials developed for community ➢ Evaluation of FDC palatability and patient preference within SHINE trial documents room for improvement ✓ Both Cap-TB and DETECT projects were pilots to adapt recommended approaches to different contexts for the purpose of enabling sustainable scale up	Align with agreed-upon implementation priorities from NTP and agenda-setting bodies and seek collaboration with COP for accelerating scale up of effective demonstrations
**Communities of Practice (COP):** Platforms for sharing best practices and lessons learned in implementation among peers; may provide expert consultation for donor and multilateral institutions	CAWG and national Child and Adolescent TB Technical Working Groups Sentinel Project Union Child and Adolescent TB Center of Excellence ➢ TB Alliance developed a platform for sharing information, lessons learned, and other updates with NTP and program staff in interested countries ✓ CaP-TB shares best practices across platform of engaged countries	Develop framework for joint responsibility across COPs for agreed-upon priorities Develop training materials on child and adolescent TB aligned with guidelines and the WHO TB knowledge-sharing platform
**Platforms for Building Political Will**		
**Civil Society and Community Engagement:** Raising awareness among general and affected populations, providing input into service delivery and program priorities, and providing support to affected communities	WHO Civil Society Taskforce WHO ENGAGE-TB Approach: Operational Guidance STP Challenge Facility for Civil Society (CFCS) Global Coalition of TB Activists ➢ Kenya’s Louder than TB Campaign engaged civil society and survivors of TB to raise awareness of the new formulation in the community	Engage with existing platforms to provide input and direction to national and local TB programs Mobilize resources for community action through existing donor programs
**Building Political Will:** Raising awareness of the burden of TB among political, opinion, and thought leaders to build political will and investment	WHO End TB Strategy UNHLM Political Declaration and 2020 UN Secretary-General progress report Annual WHO Global Tuberculosis Reports Global TB Caucus Rome Action Plan on Pediatric HIV and TB Dedicated funding for TB prevention and care, from major donors such as the Bill and Melinda Gates Foundation, CDC, the Global Fund, Unitaid and the United States Agency for International Development (USAID) as well as bilateral donors, philanthropists and others ➢ TB Alliance and partners worked with policymakers at all levels to raise awareness of the need of new FDC	Continue to build political will for addressing child and adolescent TB Monitor progress against commitments and targets

➢ Indicates an action taken in development, delivery, adaptation, and scale up of pediatric formulation of drug-susceptible TB; ✓ Indicates an action taken in development, delivery, adaptation, and scale up of decentralized models of care, including in Uganda.

## Data Availability

Not applicable.

## References

[1] WHO (2006). Guidance for National Tuberculosis Programmes on the Management of Tuberculosis in Children.

[2] Sandgren A., Cuevas L., Dara M., Gie R., Grzemska M., Hawkridge A., Hesseling A.C., Kampmann B., Lienhardt C., Manissero D. (2012). Childhood tuberculosis: Progress requires an advocacy strategy now. Eur. Respir. J..

[3] Dodd P., Gardiner E., Coghlan R., Seddon J. (2014). Burden of childhood tuberculosis in 22 high-burden countries: A mathematical modeling study. Lancet Glob. Health.

[4] WHO (2012). Global Tuberculosis Report.

[5] WHO (2013). Roadmap for Childhood TB: Toward Zero Deaths.

[6] United Nations (2018). Political Declaration of the High-Level Meeting of the United Nations General Assembly on the Fight against Tuberculosis.

[7] WHO (2018). Roadmap towards Ending TB in Children and Adolescents.

[8] Pediatric HIV Action Plan. https://www.paediatrichivactionplan.org.

[9] WHO (2022). WHO Consolidated Guidelines on Tuberculosis. Module 5: Management of Tuberculosis in Children and Adolescents.

[10] WHO (2021). Global Tuberculosis Report.

[11] Dodd P.J., Yuen C.M., Sismanidis C., Seddon J.A., Jenkins H.E. (2017). The global burden of tuberculosis mortality in children: A mathematical modelling study. Lancet Glob. Health.

[12] Marais B.J., Gie R.P., Schaaf H.S., Beyers N., Donald P.R., Starke J.R. (2006). Childhood pulmonary tuberculosis: Old wisdom and new challenges. Am. J. Respir. Crit. Care.

[13] Swaminathan S., Rekha B. (2010). Pediatric tuberculosis: Global overview and challenges. Clin. Infect. Dis..

[14] Schaaf H.S., Marais B.J., Whitelaw A., Hesseling A.C., Eley B., Hussey G.D., Donald P.R. (2007). Culture-confirmed childhood tuberculosis in Cape Town, South Africa: A review of 596 cases. BMC Infect. Dis..

[15] Marais B.J., Gie R.P., Schaaf H.S., Hesseling A.C., Obihara C.C., Starke J.J., Enarson D.A., Donald P.R., Beyers N. (2004). The natural history of childhood intra-thoracic tuberculosis: A critical review of literature from the pre-chemotherapy era. Int. J. Tuberc. Lung Dis..

[16] Jenkins H.E., Yuen C.M., Rodriguez C.A., Nathavitharana R.R., McLaughlin M.M., Donald P., Marais B.J., Becerra M.C. (2017). Mortality in children diagnosed with tuberculosis: A systematic review and meta-analysis. Lancet Infect. Dis..

[17] Brigden G., Furin J., Gulik C.V., Marais B. (2015). Getting it right for children: Improving tuberculosis treatment access and new treatment options. Expert Rev. Anti. Infect. Ther..

[18] Stop TB Partnership (2021). A Deadly Divide: TB Commitments vs. TB Realities.

[19] United Nations (2020). Progress towards the Achievement of Global Tuberculosis Targets and Implementation of the Political Declaration of the High-Level Meeting of the General Assembly on the Fight against Tuberculosis: Report of the Secretary-General.

[20] Reuter A., Seddon J.A., Marais B.J., Furin J. (2020). Preventing tuberculosis in children: A global health emergency. Paediatr. Respir. Rev..

[21] Martinez L., Cords O., Horsburgh C.R., Andrews J.R. (2020). The risk of tuberculosis in children after close exposure: A systematic review and individual-participant meta-analysis. Lancet.

[22] Dodd P.J., Yuen C.M., Becerra M.C., Revill P., Jenkins H.E., Seddon J.A. (2018). Potential effect of household contact management on childhood tuberculosis: A mathematical modelling study. Lancet Glob. Health.

[23] President’s Emergency Plan for AIDS Relief, Monitoring, Evaluation, and Reporting Dataset, TB Screening and TPT: Global.

[24] Fox G.J., Barry S.E., Britton W.J., Marks G.B. (2013). Contact investigation for tuberculosis: A systematic review and meta-analysis. Eur. Respir. J..

[25] Njie G.J., Morris S.B., Woodruff R.Y., Moro R.N., Vernon A.A., Borisov A.S. (2018). Isoniazid-Rifapentine for Latent Tuberculosis Infection: A Systematic Review and Meta-analysis. Am. J. Prev. Med..

[26] WHO (2018). Latent Tuberculosis Infection: Updated and Consolidated Guidelines for Programmatic Management.

[27] Tuberculosis Clinical Trials Consortium Study 35. https://clinicaltrials.gov/ct2/show/NCT03730181.

[28] Dodd P.J., Sismanidis C., Seddon J.A. (2016). Global burden of drug-resistant tuberculosis in children: A mathematical modelling study. Lancet Infect. Dis..

[29] WHO (2014). Guidance for National Tuberculosis Programmes on the Management of Tuberculosis in Children: Second Edition.

[30] Swaminathan S., Ramachandran G. (2015). Challenges in childhood tuberculosis. Clin. Pharmacol. Ther..

[31] WHO (2021). Who Consolidated Guidelines on Tuberculosis: Module 2: Screening.

[32] WHO (2021). WHO Consolidated Guidelines on Tuberculosis. Module 3: Diagnosis—Rapid Diagnostics for Tuberculosis Detection 2021 Update.

[33] Faust L., Abdi K., Davis K., He C., Mehrotra C., Stibolt E. (2019). The Roll-out of Child-friendly Fixed-dose Combination TB Formulations in High-TB-Burden Countries: A Case Study of STEP-TB. J. Epidemiol. Glob. Health.

[34] Gumbo T., Makhene M.K., Seddon J.A. (2016). Partnerships to Design Novel Regimens to Treat Childhood Tuberculosis, Sui Generis: The Road Ahead. Clin. Infect. Dis..

[35] Usherenko I., Basu Roy U., Mazlish S., Liu S., Benkoscki L., Coutts D., Epstein S., Qian M., Rafiq S., Scott C. (2015). Pediatric tuberculosis drug market: An insider perspective on challenges and solutions. Int. J. Tuberc. Lung Dis..

[36] Burman W.J., Cotton M.F., Gibb D.M., Walker A.S., Vernon A.A., Donald P.R. (2008). Ensuring the Involvement of Children in the Evaluation of New Tuberculosis Treatment Regimens. PLoS Med..

[37] Hoagland D., Zhao Y., Lee R.E. (2016). Advances in Drug Discovery and Development for Pediatric Tuberculosis. Mini. Rev. Med. Chem..

[38] McKenna L., Frick M., Seaworth B., Furin J. (2015). The ‘invisibility’ of children with tuberculosis. J. Publ. Health Policy.

[39] WHO (2021). Report of the Meeting to Review the Paediatric Antituberculosis Drug Optimization Priority List.

[40] Garcia-Pratts A. SS09 Best practices in pediatric DR-TB treatment—Current Management Strategies. Proceedings of the Union World Confernece on Lung Health.

[41] McAnaw S.E., Hesseling A.C., Seddon J.A., Dooley K.E., Garcia-Prats A.J., Kim S., Jenkins H.E., Schaaf H.S., Sterling T.R., Horsburgh C.R. (2017). Pediatric multidrug-resistant tuberculosis clinical trials: Challenges and opportunities. Int. J. Infect. Dis..

[42] President’s Emergency Plan for AIDS Relief (2021). 2021 Country Operational Plan Guidance for All Pepfar Countries.

[43] WHO (2022). WHO Operational Handbook on Tuberculosis. Module 5: Management of Tuberculosis in Children and Adolescents.

[44] WHO (2019). Multisectoral Accountabilty Framework to Accelerate Progress to End Tuberculosis by 2030.

[45] WHO (1994). WHO Tuberculosis Programme: Framework for Effective Tuberculosis Control.

[46] WHO (2003). Treatment of Tuberculosis: Guidelines for National Programmes.

[47] WHO (2010). Rapid Advice: Treatment of Tuberculosis in Children.

[48] (2017). New Pathways for Childhood TB Treatment: Lessons from the STEP-TB Project.

[49] WHO (2016). Technical Briefing Note: Technical Step Process to Switch to New Paediatric Tuberculosis Formulations.

[50] Maleche-Obimbo E., Wanjau W., Kathure I. (2015). The journey to improve the prevention and management of childhood tuberculosis: The Kenyan experience. Int. J. Tuberc. Lung Dis..

[51] TB Alliance TB Alliance Child Friendly Medications. https://www.tballiance.org/child-friendly-medicines.

[52] Kaiser B. Setting the scene for development of child-friendly rifapentine—Lessons learned from other child-friendly formulations. Proceedings of the 52nd Union World Conference on Lung Health.

[53] WHO (2021). WHO Essential Medicine List for Children (EMLc).

[54] WHO (2017). Guidelines for the Treatment of Drug-Susceptible TB and Patient Care.

[55] WHO (2021). Updated Recommendations on Service Delivery for the Treatment and Care of People Living with HIV.

[56] Yuen C.M. The impact of different care models of child and adolescent TB diagnostic, treatment and prevention outcomes-systematic review. Proceedings of the Union World Conference on Lung Health.

[57] Casenghi M. Closing the paeditaric TB detectiongap: Where can we find the missing children?. Proceedings of the Union World Conference on Lung Health.

[58] Berset M. Implementing TPT for Children: Experiences from CaP-TB Project in Nine Sub-Saharan African Countries. Proceedings of the Union World Conference on Lung Health.

[59] Tran C., Moore B.K. (2021). 17b Considerations for Incorporating TPT into Differentiated Service Delivery Models—Child and Adolescent Populations. https://www.pepfarsolutions.org/s/Considerations-for-TPT-in-DSD-for-children-and-adolescents_Final_19Nov2021.docx.

[60] Zawedde-Muyanja S., Nakanwagi A., Dongo J., Sekadde M., Nyinoburyo R., Ssentongo G., Detjen A.K., Mugabe F., Nakawesi J., Karamagi Y. (2018). Decentralisation of child tuberculosis services increases case finding and uptake of preventive therapy in Uganda. Int. J. Tuberc. Lung Dis..

[61] Dongo J.P., Graham S.M., Nsonga J., Wabwire-Mangen F., Maleche-Obimbo E., Mupere E., Zawedde-Muyanja S. (2021). Implementation of an Effective Decentralised Programme for Detection, Treatment and Prevention of Tuberculosis in Children. Trop. Med. Infect. Dis..

[62] (2016). DETECT Child TB Project: Report of an External Evaluation.

[63] Sekadde M. Scaling up the experieces from the DETECT TB (DEcentralise TB services and Engage Communities to Trasnform lives of Children with TB) approach in Uganda. Proceedings of the Union World Conference on Lung Health.

[64] Heldal E., Dlodlo R.A., Mlilo N., Nyathi B.B., Zishiri C., Ncube R.T., Siziba N., Sandy C. (2019). Local staff making sense of their tuberculosis data: Key to quality care and ending tuberculosis. Int. J. Tuberc. Lung Dis..

[65] Lungu P. Impact of COVID-19 on TB/HIV Programmes in Zambia. Proceedings of the International AIDS Society Conference on HIV Science.

[66] Schwoebel V., Koura K., Adjobimey M., Gnanou S., Wandji A., Gody J.-C., Delacourt C., Detjen A., Graham S.M., Mass-erey E. (2020). Child contact investigation and TPT: How to operationalise normative guidance in real life in resource-limited settings and increase uptake of TPT and TPT completion. Int. J. Tuberc. Lung Dis..

